# “Impossible” Somatosensation and the (Ir)rationality of Perception

**DOI:** 10.1162/opmi_a_00040

**Published:** 2021-07-06

**Authors:** Isabel Won, Steven Gross, Chaz Firestone

**Affiliations:** Department of Psychological and Brain Sciences, Johns Hopkins University, Baltimore, MD, USA; Department of Cognitive Science, Johns Hopkins University, Baltimore, MD, USA; Department of Psychological and Brain Sciences, Johns Hopkins University, Baltimore, MD, USA; Department of Cognitive Science, Johns Hopkins University, Baltimore, MD, USA; Department of Philosophy, Johns Hopkins University, Baltimore, MD, USA; Department of Psychological and Brain Sciences, Johns Hopkins University, Baltimore, MD, USA; Department of Cognitive Science, Johns Hopkins University, Baltimore, MD, USA; Department of Philosophy, Johns Hopkins University, Baltimore, MD, USA

**Keywords:** perception, somatosensation, impossible figures, magic, rationality

## Abstract

Impossible figures represent the world in ways it cannot be. From the work of M. C. Escher to any popular perception textbook, such experiences show how some principles of mental processing can be so entrenched and inflexible as to produce absurd and even incoherent outcomes that could not occur in reality. However, impossible experiences of this sort are mostly limited to visual perception; are there “impossible figures” for other sensory modalities? Here, we import a known magic trick into the laboratory to report and investigate an impossible experience for somatosensation—one that can be physically felt. We show that, even under full-cue conditions with objects that can be freely inspected, subjects can be made to experience a single object alone as feeling heavier than a group of objects that includes the single object as a member—an impossible and phenomenologically striking experience of weight. Moreover, we suggest that this phenomenon—a special case of the size-weight illusion—reflects a kind of “anti-Bayesian” perceptual updating that amplifies a challenge to rational models of perception and cognition.

## INTRODUCTION

**Figure 1.**
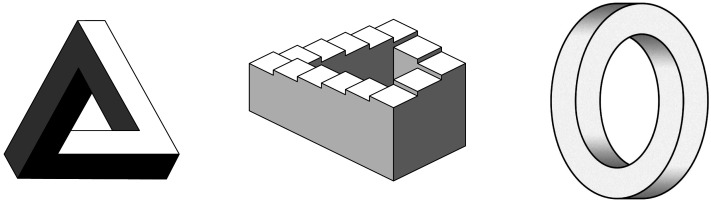
**Examples of images that produce “impossible” perceptual experiences.** Such figures represent the world in ways it could not be (e.g., a triangle with three 90° angles).

One of the most striking and puzzling aspects of our minds is that they permit “impossible” experiences: perceptions of the world that are physically, geometrically, or even conceptually incoherent. For example, when an image carefully exploits patterns of shading and layout, we may see it as a triangle with three 90° angles, or as a closed staircase that descends in every direction (Figure 1), even though such objects could never actually exist. Impossible experiences are intuitively compelling, but they are also theoretically significant: They go beyond ordinary visual illusions (e.g., when stimuli appear larger, faster, or darker than they really are) in revealing how some principles of mental processing can be so entrenched and inflexible as to produce absurd and even self-contradictory outcomes that could not occur in reality. Moreover, they do so in a way that is intrinsic to the perceiver’s experience itself, such that they also go beyond (a) inconsistencies in the underlying perceptual processing (e.g., Smeets & Brenner, 2008, 2019; Sousa et al., 2011) and (b) conflicts between perception and higher level knowledge (Firestone & Scholl, 2016a). In many impossible figures, a single experience represents the world in a way it cannot be.

Despite their ubiquity and popularity, the scope and impact of impossible experiences have been limited in at least two important ways. First, whereas more conventional perceptual illusions are found in all sensory modalities, impossible figures arise mostly or only within visual perception (with perhaps an exception in audition; Shepard, 1964); indeed, it may be difficult to even fathom impossible experiences for other senses (e.g., an impossible taste or smell). Second, all known impossible figures require unnatural or impoverished viewing conditions, such as stimuli drawn on two-dimensional surfaces, or accidental views of precisely arranged 3D scenes (Deregowski, 1969; Macpherson, 2010; Penrose & Penrose, 1958). Indeed, these factors may have diminished the perceived scientific value of such phenomena, casting them as ecologically invalid tricks rather than theoretically significant data for understanding the mind.

### Impossible Feelings?

In contrast to these classical examples, here we report an impossible perceptual experience that can be *physically felt* (rather than seen or heard), using ordinary real-world objects that perceivers can freely inspect (rather than restrictive presentation conditions). We suggest that the space of impossible experiences is larger than has been appreciated, extending into another sensory modality. Importantly, we further suggest that the present phenomenon is not just a phenomenological curiosity, but rather that it interacts with discussions about “rational” processing in the mind by amplifying a prominent challenge to Bayesian models of perception and cognition. Finally, we connect this phenomenon to a research trend where principles known to professional magicians can inform psychological investigation (Ekroll et al., 2017; Rensink & Kuhn, 2015).

### The Present Experiments

Our work here exploits the logic of the century-old size-weight illusion (Charpentier, 1891). In the classical incarnation of this illusion, subjects are shown two objects whose sizes differ but whose weights are identical; surprisingly, subjects who lift both objects find that the smaller object feels heavier than the larger object. Though the cognitive mechanisms underlying this illusion have remained surprisingly difficult to pin down, the conditions under which it occurs are extensively catalogued, including variants of the illusion arising even in blind or blindfolded subjects who sense the objects’ sizes using only touch (Ellis & Lederman, 1993) or even echolocation (Buckingham et al., 2015). In these and many other cases, a smaller object feels heavier than an equally weighted larger object (for a review, see Buckingham, 2014).

The experience elicited by the classical size-weight illusion is certainly odd (and even improbable), but it is not quite “impossible”; after all, the smaller object could be (and usually is) made of a denser material than the larger object. But might there be a way to modify this illusion in ways that truly do produce an impossible—and even conceptually incoherent—percept? Indeed, a lesser-known “bar trick” variant of the illusion might do just that. Instead of comparing one object to an unrelated object, this variant asks subjects to compare one object to a *group* of objects that includes the first object as a member. The thought is that, under these circumstances, subjects might perceive the single object alone as heavier than a group including the single object. Of course, a group of objects could never weigh less than a member of that group; and so if this is indeed what subjects experience, they will have had an impossible experience of weight.^1^

To our knowledge, this latter phenomenon, if it occurs at all, has never been reported in the scientific literature.^2^^,^^3^ We thus (a) introduce it here, (b) run several new experiments exploring it (and controlling for alternative explanations that have never been discussed or tested), and (c) suggest that it poses a unique challenge to rational models of perception and cognition, due to the chain of updating that seems to occur in producing it. We expand on this final possibility in the General Discussion, since the nature of this challenge is clearest only after the experiments are described.

## EXPERIMENT 1: IMPOSSIBLE SOMATOSENSATION

### Methods

All of the raw data supporting the experiments in this article are available in a Materials Archive See: https://osf.io/g7kb8. We report how we determined our sample size, all data exclusions, all manipulations, and all measures in the study.

#### Participants

Thirty subjects were recruited from the Johns Hopkins University (JHU) community. Given how subjectively apparent the effect was to each author of this article, we chose this sample size because it would produce a statistically reliable effect if more than two-thirds of subjects experienced the illusion. All other experiments here used this sample size.

#### Stimuli and Procedure

**Figure 2.**
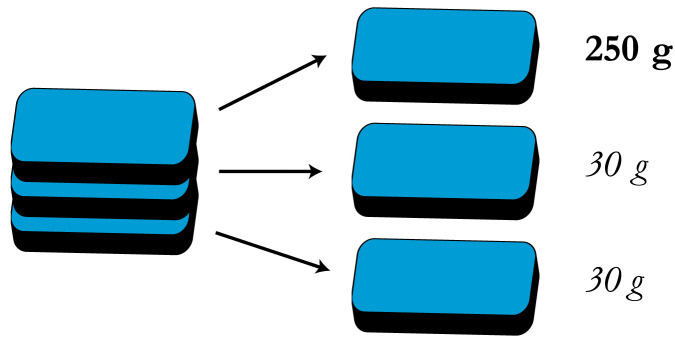
**Schematic depiction of the three-boxes illusion.** In the present experiments, subjects performed lifts of identical-looking boxes. Though the boxes were identical in appearance, one of the boxes weighed much more than the others. Subjects lifted either the heavy box alone, or all three together.

Subjects saw three identical-looking opaque boxes in a stack, which we refer to here as Boxes A, B, and C. All three boxes were 6.7 cm × 8.7 cm × 1.6 cm, 3D-printed in blue ABS plastic. Instructions for creating these boxes are available in our materials archive.^4^ Unbeknownst to subjects, Box A (located on top of the stack) was filled with zinc (250 g), while Boxes B and C were empty (weighing only 30 g; Figure 2).

Subjects performed two lifts, one immediately after the other. In one case, they lifted Boxes A, B, and C together; in another case, they lifted Box A alone. Here in Experiment 1, subjects lifted the boxes simply by grasping them with their hands, in whatever posture felt natural; later experiments varied this grasp posture.

After the two lifts (order counterbalanced across subjects), subjects were asked which lift felt heavier (or, for half of subjects, which lift felt lighter), and the experimenter recorded the subject’s response.

### Results

**Figure 3.**
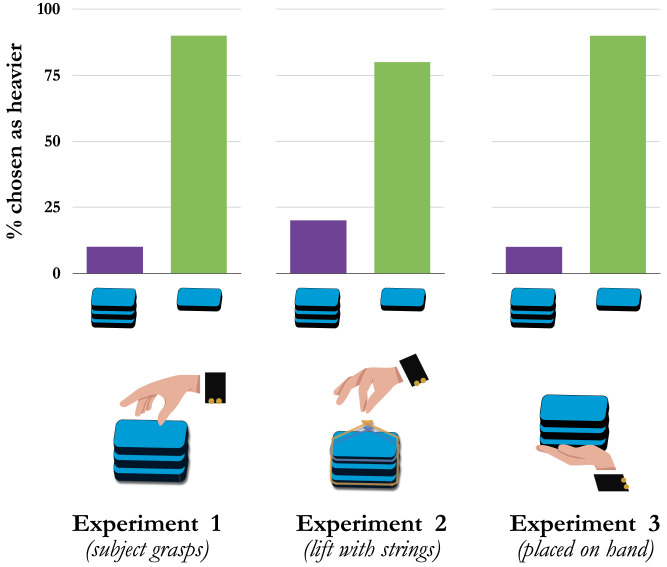
**Results from Experiments 1–3.** No matter how subjects lifted the boxes, they overwhelmingly reported that the single heavy box seem to weigh more than all three boxes together—an “impossible” experience of weight. (Though the image for Experiment 3 shows a “floating” hand, subjects in that experiment in fact passively rested their hands on a flat table.)

Subjects overwhelmingly reported that Box A alone felt heavier than Boxes A, B, and C together (90% of subjects reporting A > ABC, binomial probability test, *p* < .001; Figure 3).^5^ However, this result should be “impossible,” because the sum of weights over a set of objects could never be *less* than the sum of weights over a subset of those objects: Unless the boxes somehow changed between lifts, Box A couldn’t weigh more than a group of weighted objects that *includes Box A as a member*.

Indeed, the experience was so striking that subjects often spontaneously and astoundedly commented on its impossibility, and even requested to lift the objects again after the session was over. Anecdotally, those subjects reported that the illusion persisted even during these repeated lifts, including when subjects placed all three boxes on their palm and then suddenly removed the two lighter boxes—distilling the phenomenon into a single impossible “moment” wherein *removing* weight caused the sensation of *adding* weight.

These results thus confirmed experimentally what is also just subjectively apparent upon casually lifting the boxes: Under these conditions, a single box alone feels impossibly heavier than a group of boxes that includes the single box as a member.

## EXPERIMENT 2: EQUATING GRASP POSTURE

An important assumption for interpreting this effect as an impossible experience is that it not be driven by differences in how subjects perform the two lifts. (This is also important for interpreting other instances of the size-weight illusion.) For example, subjects in Experiment 1 inevitably used a different grasp posture when lifting all three boxes (ABC) vs. when lifting just one (A), since the ABC lift required fitting three times as much material in their hands, and so required a wider grasp, more extended fingers, and other such adjustments. Could this sort of difference explain the difference in perceived weight? Experiment 2 ruled out this possibility by attaching handle-like “tabs” to the boxes, such that each lift was performed by pinching a single tab between one’s fingers. If the effect persists under these circumstances, then it is unlikely to be explained by differences in grasp posture, since the same posture was assumed for both lifts.

### Methods

Experiment 2 was identical to Experiment 1 except as noted here. Thirty new subjects participated. This time, two loops of clear fishing line were attached to the boxes—one loop around all three boxes (Boxes ABC), and one loop around just the heavier box (Box A), with each ending in a small “tab” secured with electrical tape. Subjects performed the same lifts as in Experiment 1, but here they simply pinched the tabs between their thumb and index fingers—and did so in the same way across the two lifts.

### Results

As in Experiment 1, subjects reported that A felt heavier than ABC (80%, *p* = .002; Figure 3), even when equating grasp posture across the lifts. This suggests that differences in hand shape or finger extension (etc.) could not explain the impossible experience of one object feeling heavier than a group that includes it as a member.

## EXPERIMENT 3: EQUATING LIFT FORCE

Despite the phenomenon appearing under naturalistic grasping conditions and under conditions of equated grasp posture, a further possibility in the previous two experiments is that subjects exerted a greater lifting force for the ABC lift than the A lift, perhaps because they expected ABC to be heavier than A. In that case, ABC might have seemed lighter than A if subjects used much more force on ABC than on A (though see Flanagan & Beltzner, 2000). Could this explain the results observed so far?

On one hand, we noted in Experiment 1 that subjects observed (anecdotally) that the illusion persisted after multiple lifting attempts; in that case, subjects should have then had the correct expectations about the boxes, which in turn should have caused them not to badly miscalibrate their lifting forces on repeated lifts. Moreover, the classical size-weight illusion is known not to depend on differences in lifting force, even if such differences are indeed observed in some cases (Buckingham, 2014).

On the other hand, even under such conditions, implicit or automatic expectations might have caused subjects to exert a greater force in one case than in another. Experiment 3 thus ruled out even this possibility, by not requiring any “lifting” at all but rather asking subjects to passively feel the weight of the boxes while their hands rested on a table.

### Methods

Experiment 3 was identical to Experiment 1 except as noted here. Thirty new subjects participated. This time, rather than grasp the boxes, the subjects placed their hands palm up on a flat table; then, the experimenter herself placed the boxes (either ABC, or A, in a counterbalanced order) onto subjects’ passively open palms. This method not only controlled for grasp posture in yet another way (since the subjects’ hands were in the same posture for both lifts), but also for lifting force, since any force exerted by subjects was small or nonexistent.

### Results

As in Experiment 1, subjects reported that A was heavier than ABC (90%, *p* < .001; Figure 3), even when they didn’t “lift” the boxes at all but simply felt their pressure against their passively open palms. This suggests that neither differences in grasp posture nor in lifting force explain the impossible experience of one object feeling heavier than a group that includes it.

## GENERAL DISCUSSION

In three experiments, subjects perceived a single object alone as heavier than a group containing that object. We interpret this experience as a somatosensory analog to “impossible figures“ that arise in visual perception.

### An “Impossible Figure” for Somatosensation

Importantly, the present phenomenon goes beyond known somatosensory illusions or unusual bodily experiences. For example, in “Aristotle’s Illusion,” touching one’s nose with crossed index and middle fingers produces the bizarre feeling of having two noses (Hayward, 2008; Lackner, 1988). Similarly, stimulation of the biceps tendon can make one feel as though one’s arm is extending (Goodwin et al., 1972)—so much so that, after enough stimulation, one’s arm would have to be so extended as to imply a break at the elbow.^6^ However, haptic illusions such as these (see also Guterstam et al., 2011; Guterstam et al., 2018), while striking and unusual, arguably lack the genuinely “impossible” character of the present phenomenon (and tend to involve only strange bodily sensations rather than interactions with other objects). In other words, sprouting a new nose or extending one’s arm past its breaking point are certainly biologically implausible experiences; but the present phenomenon is not merely strange or unusual—it is physically or even conceptually *incoherent*.

It is this incoherence that we emphasize here, since in our view it is what makes this phenomenon a somatosensory analog to the impossible visual figures in Figure 1. Just as those images show a kind of global incoherence (even though many regions of the images are locally coherent), the present experience has a similar character. And although the present phenomenon does rely on being temporally extended (with the lifts occurring one after another but not literally at the same time), this is arguably the case even for impossible visual figures, which are difficult to take in all at once. For example, in the “impossible staircase” (Figure 1, center), one may find one’s attention flitting around the staircase until one notices that the local transitions add up to an impossible global figure. Moreover, given that it is possible in the present phenomenon to visibly reduce the weight of the stack (by removing B and C) and yet cause a perceived *increase* in weight, it may well be that the present phenomenon can be distilled into a single impossible “moment,” and perhaps thus a single impossible experience.^7^

Note further that it was not a foregone conclusion that the size-weight illusion would extend to the present case. An alternative possibility, for example, was that somatosensory processing would have access to part/whole relations in held objects (in ways analogous to part-based decomposition in visual perception; Hoffman & Richards, 1984; Lowet et al., 2018; Singh et al., 1999), and that heaviness perception would respect various physical constraints on such part-whole relations. The fact that this did not occur may suggest that haptic processing does not segment objects into parts in the same way as visual processing does (or at least may not incorporate physical constraints into whatever part-based segmentation it does carry out, or cannot carry forward such information for use even across extremely short time-scales, etc.).

### More Than a Trick

Beyond adding to the inventory of impossible perceptual experiences, the fact that our minds generate such an impossible or incoherent outcome in the present case may interact with classical and contemporary discussions about the “rationality” of mental processing, in at least two ways.

#### “Anti-Bayesian” Updating

First, the classical size-weight illusion—where smaller objects feel heavier than equally-weighted larger objects—has sometimes been described as defying Bayesian norms of inference (Brayanov & Smith, 2010; Buckingham & Goodale, 2013). Typically, the mind’s interpretation of uncertain data is *attracted toward* its priors, in line with a broadly Bayesian recommendation. For example, if one encounters an object whose circular retinal image is equally consistent with it being (a) a sphere or (b) an elongated ellipsoid viewed at just the right angle to project a circle to the viewer, one typically experiences that object as a sphere—arguably because this experience reflects the visual system’s prior assumptions about which shapes and views are most likely. In other words, ambiguity in the data is resolved “in favor” of the assumptions one had prior to encountering those data. By contrast, the size-weight illusion instead seems to involve *repulsion from* such priors: One comes in expecting the larger object to be heavier than the smaller object, and then one receives equivocal or ambiguous sensory evidence about which is truly heavier (since they really weigh the same); but then one somehow experiences the larger object as *lighter* than the equally weighted smaller object. In other words, the mind seems to resolve the ambiguous sensory evidence “against” the larger-is-heavier prior, rather than toward it—an apparent counterexample to notions that perception and cognition implement or approximate Bayesian inference.

The present results, it seems to us, amplify this challenge further, and make the “irrationality” of this pattern of updating all the more stark. Evidently, the norm-defying bias in the size-weight illusion is so powerful that it can generate not only improbable outcomes (as in the classical size-weight illusion, as well as in other phenomena sometimes described as as anti-Bayesian; Wei & Stocker, 2015; see also Rahnev & Denison, 2018) but also impossible outcomes whose probability should be zero and that should therefore be unacceptable to a rational updater—since no chain of updating should end with A being heavier than ABC (see also Mandelbaum, 2019; Mandelbaum et al., 2020).

Indeed, the astonishment that subjects express upon experiencing this phenomenon—both here in our study, as well as in Koseleff (1937), where subjects described their experience as “*unlogisch*”—seems to tell against other rationality-preserving accounts of this illusion. For example, one classical account of the size-weight illusion suggests that subjects are substituting or integrating density with weight (Ross & Di Lollo, 1970; perhaps also Stevens & Rubin, 1970). In that case, it would be natural (and perfectly in line with Bayesian norms) to experience the larger object as lighter, if “lighter” here roughly means “less dense” rather than “less heavy.” But such an account seems less plausible for the present phenomenon: If subjects were reporting the objects’ densities, rather than their weights, then there should be no reason for subjects to find their experiences here “impossible” or “illogical”—since there is nothing impossible about a single object being denser than other members of its group, or a stack of objects feeling less dense together than one of its members feels alone. By contrast, it is indeed impossible for a single object to be heavier than a group of which it is a member—and so the present results provide a new kind of evidence for the (anti-Bayesian) weight-based account of the size-weight illusion, by better aligning with the explicit reports of subjects who experience it.^8^

Of course, the nature of this challenge is controversial, and a full account of it is beyond the scope of the present discussion. Indeed, there are now more sophisticated density-based accounts exploring how the mind might rationally estimate the relative weight of two objects by first estimating their densities (about which the perceiver might have prior hypotheses) and then integrating that estimate with the objects’ perceived sizes. For example, Peters et al. (2016) explore such an account, and argue that the size-weight illusion is not anti-Bayesian after all. (It is unclear, however, how their model—which directly generates judgments of heaviness ratios—applies to sequential liftings with temporarily separated haptic signals and heaviness percepts, as in the present experiments.) More generally, for any such model (including the more recent approach proposed by Lieder & Griffiths, 2020; see also Wei & Stocker, 2015), the crux of the present challenge is for it to recast as “rational” not only improbable outcomes, but also impossible ones.

#### A Perceptual “Conjunction Fallacy”?

Another intriguing aspect of the present results is that they are reminiscent of the “conjunction fallacy” from the heuristics and biases tradition, wherein two propositions jointly seem more probable than one of the propositions alone (Tversky & Kahneman, 1983). For example, when told a story about a young woman (“Linda”) who majored in philosophy and is concerned with social justice, subjects judge it less likely that Linda is a bank teller than that she is a bank teller and active in the feminist movement. But this is impossible, since a conjunction of propositions could never be more probable than one of the propositions making up the conjunction. The present phenomenon, wherein a “conjunction” of objects feels lighter than one “conjunct,” has a similar flavor, since it is also true that a collection of objects could not be less massive than one of the objects making up the collection. In that case, these results could suggest that it is not only higher level cognition but also perception itself that can systematically fail when considering together entities that are usually considered separately (and in a way that goes beyond even the visual images in Figure 1, which are incoherent in their own way but do not readily evoke the conjunction fallacy—since they are not cases where “adding” or “joining” one thing to another moves the relevant representation in the “wrong” direction).

This interpretation of the present phenomenon would not only be theoretically interesting in its own right (since it was not previously thought that perception itself might “commit” this type of error), but it could also matter for other questions about the perceptual or cognitive nature of various psychological phenomena. For example, Ludwin-Peery et al. (2020) recently discovered that intuitive physical reasoning (e.g., about the movement of physically interacting objects) exhibits conjunction-fallacy-like behavior, and interpreted this as evidence that physical intuitions must have a cognitive basis rather than a perceptual one (cf. Firestone & Scholl, 2016b, 2017; Hafri & Firestone, 2021; Little & Firestone, 2021)—because perception isn’t the sort of process that could arrive at such a fallacious outcome. The present work suggests that this may not be a secure inference, if perception can indeed show conjunction-fallacy-like behavior after all. Of course, the phenomenon we explore here replaces probability (in the Linda case) with weight (in the present case), but nevertheless the structural similarity of these two cases seems at least to leave open the question of whether perception is immune to such fallacious patterns of updating.

One might object to the analogy between the present phenomenon and the conjunction fallacy by noting that the present phenomenon is simply a natural extension of whatever perceptual heuristics are operative in the size-weight illusion, rather than the discovery of a new phenomenon unto itself. However, in our view such an analysis only *increases* the appropriateness of the analogy. The conjunction fallacy, after all, is itself not typically considered a single phenomenon but rather a special case arising from the application of heuristics that are operative in many other circumstances—especially the representativeness heuristic, according to which the probability of an event is judged based on its similarity to the process that generated it, or to a family of related events. On a standard account of the conjunction fallacy, it arises because “Linda the feminist bank teller” seems more representative of her biography than “Linda the bank teller”; and this sense of representativeness is so powerful a heuristic that it can lead people to draw flatly impossible conclusions when just the right scenario is set up. Normally, of course, representativeness-based reasoning doesn’t lead one to reach fallacious conclusions, just as normally whatever heuristics give rise to the size-weight illusions don’t produce impossible perceptual experiences. In other words, both the conjunction fallacy and the present phenomenon of impossible somatosensation arise from heuristics that are unproblematic when applied locally (e.g., to one proposition, or to one object) but can become “impossible” when applied globally (e.g., when comparing a conjunction of propositions against one of its conjuncts, or when comparing a group of objects to one of its members) in certain conditions.

### From Magic to Mind

Finally, our results add to a growing literature that has taken inspiration from professional magic to study and reveal principles of psychological processing. For example, previous work along these lines has applied specific insights from magicians’ knowledge of misdirection to shed light on the operation of visual attention, leading to discoveries of new phenomena of change blindness (Yao et al., 2019). The same is true of more specific magical “demonstrations,” including powerful and previously unknown consequences of amodal completion (Ekroll et al., 2016). Our work here is another example of this growing trend, and so further suggests that magic can be a source of meaningful insight into how our minds work (for reviews, see Ekroll et al., 2017; Macknik et al., 2008).

More generally, our results show how impossibility can be not only seen and heard, but also felt—and in ways that matter for core questions about mental processing.

## ACKNOWLEDGMENTS

For helpful discussion and/or comments on previous drafts, we thank Simon Brown, Ernie Davis, Jorge Morales, Ian Phillips, Jeroen Smeets, and members of the JHU Perception and Mind Laboratory. For technical support, we thank Mark Fuller.

## FUNDING INFORMATION

This work was supported by NSF BCS-2021053 awarded to CF, as well as a JHU Science of Learning Institute Grant to SG and CF.

## AUTHOR CONTRIBUTIONS

IW: Conceptualization: Equal. Data curation: Lead; Formal Analysis; Lead; Investigation: Lead; Methodology: Lead; Visualization: Lead Writing – original draft: Equal Writing – review & editing: Equal. SG: Conceptualization: Equal; Funding acquisition: Supporting; Supervision: Supporting; Writing – original draft: Supporting. Writing – review & editing: Supporting. CF: Conceptualization: Equal; Data curation: Supporting; Formal Analysis: Supporting; Funding acquisition: Lead; Investigation: Supporting; Methodology: Supporting; Supervision: Lead; Visualization: Supporting; Writing – original draft: Equal; Writing – review & editing: Equal.

## Notes

^1^ Philosophers distinguish various kinds of impossibility. For example, logical impossibility is formal inconsistency (of the form “P and not-P”); conceptual impossibility is inconsistency with the meaning of the concepts deployed (“He is a married bachelor”); nomological impossibility is inconsistency with natural law (“It traveled faster than the speed of light”) (Kment, 2021). It is often controversial what kind of impossibility is at issue in a particular case. For our purposes, it suffices that it is at least nomologically impossible, at any given time, for a part to weigh more than the whole of which it is a part—and, further, nomologically impossible, across times, for a part to weigh more than the whole of which it is a part if neither has changed in any relevant way.^2^ The closest reference, perhaps, is Koseleff (1937), who reported that a small heavy block lifted alone felt heavier than when lifted with the addition of a much larger (but lighter) block on top—such that subjects described their experience as “*unlogisch*” in debriefing. However, this older study included significant visual differences between the objects (unlike in our studies here), did not counterbalance lift order (with the two objects always lifted together before the single object, which could produce effects of adaptation or hysteresis), and failed to equate grasp posture and force across the lifts. (Indeed, the same paper reported that the effect was reduced or eliminated when subjects pushed the boxes up from below vs. when they were grasped from above.) Finally, this previous report of course did not connect the findings up with contemporary issues around Bayesian models of perception and cognition (as we do; see General Discussion). Still, this result is encouraging enough to suggest that a modernized version might produce similar or even more striking results, and in ways that could justify the weightier theoretical consequences we attach to it here. We thank Robert Volcic for bringing this paper to our attention.^3^ A variant of it, however, is discussed (and occasionally sold) as a “magic trick” (https://www.grand-illusions.com/three-card-box-illusion-c2x21140225).^4^ We thank Mark Fuller for assistance designing and creating these objects.^5^ Two subjects insisted that the two lifts felt the “same.” When this happened, we coded their response as 0.5 for the purposes of the binomial probability test (rather than 1 or 0). However, no result here—both in this experiment and later experiments—depended on this coding scheme; in other words, all effects remain statistically reliable even if those data are simply excluded, and even if they are counted *against* our hypothesis.^6^ There are also variants of the size-weight illusion that approach the present result but do not imply the same consequences. For example, a single barbell-shaped object whose width (but not weight) can be physically adjusted may feel differently light or heavy depending on its size (Plaisier & Smeets, 2015), even though the same amount of material is seen. This phenomenon too may be viewed as “impossible” in some sense, since it is not physically possible for an object to get heavier simply by changing its shape (though it is possible for shape changes to produce differences in leverage and required lifting effort). However, this result still lacks the kind of conceptual *incoherence* of the phenomenon we explore here, and also doesn’t evoke the “conjunction fallacy” (nor is it presented as such) in the way the present phenomenon does. See the main text for an expansion on this latter point.^7^ *Still*, it may nonetheless be objected that, insofar as this experience is temporally extended, it is not an experience of something *impossible*. According to this objection, what is impossible is that, at a specific time *t*, A weigh more than ABC; but what’s not impossible is that ABC at *t*_1_ weigh less than A at *t*_2_, since weights can change over time. A reply to this objection, however, is that it is indeed impossible that ABC at *t*_1_ weigh less than A at *t*_2_ *if* A has not changed in any relevant way—which is the case here (and is believed by the subject to be the case).^8^ A related account suggests that heaviness impressions in the size-weight illusion are better understood as impressions of how easily an object can be thrown (Zhu & Bingham, 2011) since smaller objects are often easier to throw than larger objects when weight is held constant. Still, even this account permits that the judgments made here are genuinely heaviness judgments, and so arguably preserves the impossible character of the experience.

## Supplemental Materials

https://osf.io/g7kb8
